# The effect of IPS-modified, an early intervention for people with mood and anxiety disorders: study protocol for a randomised clinical superiority trial

**DOI:** 10.1186/1745-6215-14-442

**Published:** 2013-12-24

**Authors:** Lone Hellström, Per Bech, Merete Nordentoft, Jane Lindschou, Lene Falgaard Eplov

**Affiliations:** 1 Copenhagen University Hospital, Research Unit, Mental Health Centre Copenhagen, Bispebjerg Bakke 23, DK-2400 Copenhagen, Denmark; 2 Psychiatric Research Unit, Mental Health Centre North Zealand, Dyrehavevej 48, 3400 Hillerød, Denmark; 3 Department 3344, Copenhagen Trial Unit, Centre for Clinical Intervention Research, Rigshospitalet, Copenhagen University Hospital, Blegdamsvej 9, DK-2100 Copenhagen Ø, Denmark

**Keywords:** Supported employment, Affective disorder, Anxiety, Competitive employment, Mentor support

## Abstract

**Background:**

Anxiety and affective disorders can be disabling and have a major impact on the ability to work. In Denmark, people with a mental disorder, and mainly non-psychotic disorders, represent a substantial and increasing part of those receiving disability pensions. Previous studies have indicated that Individual Placement and Support (IPS) has a positive effect on employment when provided to people with severe mental illness. This modified IPS intervention is aimed at supporting people with recently diagnosed anxiety or affective disorders in regaining their ability to work and facilitate their return to work or education.

**Aim:**

To investigate whether an early modified IPS intervention has an effect on employment and education when provided to people with recently diagnosed anxiety or affective disorders in a Danish context.

**Methods/Design:**

The trial is a randomised, assessor-blinded, clinical superiority trial of an early modified IPS intervention in addition to treatment-as-usual compared to treatment-as-usual alone for 324 participants diagnosed with an affective disorder or anxiety disorder living in the Capital Region of Denmark. The primary outcome is competitive employment or education at 24 months. Secondary outcomes are days of competitive employment or education, illness symptoms and level of functioning including quality of life at follow-up 12 and 24 months after baseline.

**Discussion:**

If the modified IPS intervention is shown to be superior to treatment-as-usual, a larger number of disability pensions can probably be avoided and long-term sickness absences reduced, with major benefits to society and patients. This trial will add to the evidence of how best to support people’s return to employment or education after a psychiatric disorder.

**Trial registration:**

NCT01721824

## Background

Anxiety and affective disorders are often associated with functional disability and can have a major impact on the ability to work [1-4]. Through the 1990s, depression alone was responsible for an annual loss of US$ 17 billion due to work absenteeism and a total cost of US$ 43.7 billion (34.8 billion Euro) each year in direct and indirect societal costs in the USA [4]. In Denmark, mental health problems account for a total of 7.3 billion Euro each year in direct and indirect societal costs [5]. Disability pension and long-term sickness absence account for the majority [5,6]. A significant amount of the total sickness absence in Denmark is due to mental illness, and disability pensions are increasingly awarded due to non-psychotic mental illness [6,7]. Hence it is crucial to start initiatives to support patients with mental health problems in retaining or regaining their employment or education.

The Individual Placement and Support (IPS)-modified, early intervention for people with mood and anxiety disorder (IPS-MA) is an individualised supported employment intervention, aiming at supporting people with recently diagnosed anxiety or affective disorders to obtain and sustain competitive employment through mentor support. It was created in 2011, based on the experience of a 1-year pilot study, aspects from the supported employment intervention IPS and findings from the literature. The method has never been investigated in a clinical trial.

A recent systematic review of randomised trials as well as controlled non-randomised cohort studies [8] found an overall lack of evidence concerning vocational rehabilitation for patients with recently diagnosed bipolar disorders, depression or anxiety disorders, but points to three important initiatives to consider: preventive interventions, return-to-work interventions and interventions concerning short- or long-term loss of employment. Preventive interventions have only been investigated for patients with depression or depressive symptoms, and show evidence in favour of individualised interventions [9-11]. Considering return to work interventions, studies suggest that an individual intervention should be combined with work-place interventions in close collaboration with mental health services [4,12,13]. Returning to work when diagnosed with depression, anxiety or bipolar disorders is also affected by personal and social factors; hence, it is important to incorporate interventions supporting these matters.

Today, vocational rehabilitation mainly consists of two different approaches: pre-vocational training, often referred to as the train-and-place model, and supported employment, referred to as place-and-train [14]. With pre-vocational training, people are trained in company internship programmes, sheltered workshops or wage-subsidised jobs before obtaining competitive employment. Supported employment aims at a rapid search for competitive employment, with on-going support after employment. In Denmark pre-vocational training is still standard.

The most intensively studied supported employment intervention is IPS [15,16], where job consultants are integrated in and act in close collaboration with the mental health services. Several randomised trials [14,17-27] have indicated that IPS is more effective in helping patients with severe mental illness obtain and sustain competitive employment compared to traditional pre-vocational training. A meta-analysis [28] of four randomised trials [29-32] found that, after 18 months, 70.4% had obtained competitive employment in the IPS group compared to 24.3% in the control group. In a review including 11 randomised trials comparing IPS to traditional pre-vocational training, 61% of the patients obtained competitive employment in the IPS group versus 23% in the control group [15]. No studies were found investigating the effect of IPS when provided to people with recently diagnosed affective or anxiety disorder. It is recommended that the intervention be modified and accommodated to psychosocial and medical aspects, and thoroughly investigated in order to show an effect when offered to, for instance, patients with recently diagnosed anxiety or affective disorders [16]. Further studies are needed in order to investigate the effect of such interventions, in addition to mental health treatment, on people’s return to work.

Sherpa ran the pilot study, from October 2010 to September 2011 (unpublished data), during which 46 patients with depression, anxiety or a bipolar disorder were referred to Sherpa from two mental health centres in Copenhagen. Two mentors and a career counsellor were employed at the time. Twenty of the participating patients had obtained either employment or education after a median of 4.2 months (range 1 to 8 months).

The above-mentioned findings from the literature, aspects from the IPS, and the experiences from the 1-year pilot study led to the creation of IPS-MA in 2011.

The IPS-MA is an individualised supported employment intervention, considering personal and social factors, as well as career counselling and financial guidance. Focus is on a rapid search for competitive employment or education, and not sheltered workshops or long internship programmes. Since people with affective disorders or anxiety are treated by either their general practitioner, psychiatric private practitioner or in mental health centres in Denmark, it is difficult to integrate IPS-MA with treatment to the same extend as in IPS. According to IPS-MA, mentors must have an assertive approach to mental health carers and social workers, and collaborate with mental health services as well as job centres and municipalities, and thereby help coordinate services provided by these.

This is the first trial comparing the effect of IPS-MA to treatment-as-usual when provided to people with recently diagnosed anxiety or affective disorders. The hypothesis is that more people receiving IPS-MA will return to work or education compared to the control group.

## Methods

### Design

**Figure 1:**
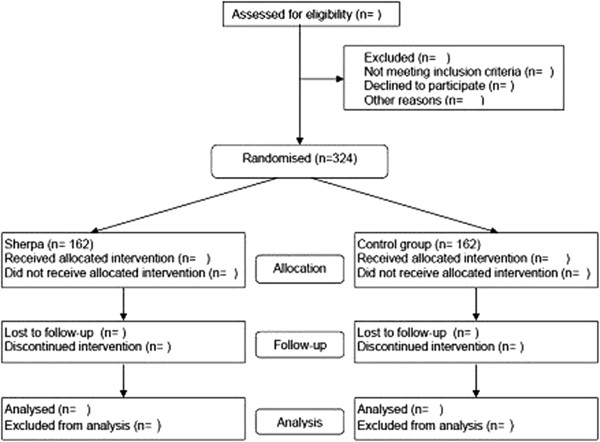
Flow chart for participants in the trial.

The Sherpa trial is a randomised, assessor-blinded, clinical superiority trial comparing IPS-MA in addition to treatment-as-usual with treatment-as-usual alone in 324 patients recently diagnosed with an affective disorder or an anxiety disorder (Figure 1).

### Participants

Participants will be recruited from Mental Health Centres and private practising psychiatrists within the Capital Region of Denmark from 1 October 2011 until 31 January 2014. Inpatients as well as outpatients are eligible.

### Inclusion criteria

Participants must be aged 18 to 60 years, diagnosed by the referring psychiatrists according to the International Classification of Diseases 10th edition criteria of affective disorders (F30-39) or anxiety disorders (F40-41), and not have had contact with mental health services for more than 3 years. They must have been employed or enrolled in education at some time during the past 2 years. They must have a pronounced wish to return to either employment or education, but not being ready to do so within the following 3 months, and equal to ‘match group’ 2 or 3. (‘Match groups’ are categories used by the job centres in Denmark [33] to estimate how far people are from the labour market. Match group 2 refers to people who can participate in pre-vocational training or courses, but who would not be able to take an ordinary job and be off social benefits within 3 months. Match group 3 refers to people with problems so severe that they cannot work or participate in pre-vocational training). Participants must have the ability to read and understand Danish, and give informed consent verbally and in writing.

### Exclusion criteria

Participants will be excluded if they have somatic co-morbidity causing reduced ability to work, primary large-scale alcohol or substance abuse, a legal guardian, forensic psychiatric arrangements, or if they do not give informed consent.

### Recruitment and randomisation

Eligible patients are informed about Sherpa, given the written information, and subsequently referred to Sherpa by their psychiatrists, nurse or social worker. A Sherpa employee calls the patient to make an appointment for inclusion and baseline interview. The assessor will interview the participants, but a Sherpa mentor will always attend the interview in order to manage the randomisation after the assessor has left and inform the participant about allocation. When a participant is included in the trial, central randomisation is performed when the Sherpa mentor calls the Copenhagen Trial Unit and gives the relevant participant information.

Randomisation is performed according to a computer-generated allocation sequence with a varying block size concealed from the investigators. The randomisation is stratified by a) four diagnoses (F31: Bipolar affective disorder; F30, F32-39: Affective disorders; F40: Phobic anxiety disorders; or F41: Other anxiety disorders), and b) two match groups (match group 2 or 3).

### Blinding

It is not possible to blind the participants, the Sherpa mentors, or career counsellors, practitioners and carers who deliver the intervention. However, they are strongly urged not to reveal the allocation to the rest of the research team. The assessor and research team will be blinded to the allocated intervention group throughout the entire trial period. Should blinding be violated, a second assessor will complete the follow-up interview. Furthermore, during statistical analyses, the two intervention groups will be coded as, for example, X and Y, and the code will not be broken until the research team has drawn two conclusions; one assuming X is the intervention group and Y is the control group, and one conclusion assuming the opposite.

### Interventions

#### The experimental intervention

Participants randomised to the Sherpa group will be offered IPS-MA in addition to treatment-as-usual (see description for the control group). A Danish protocol describing the IPS-MA method can be acquired by contacting the corresponding author. An English version is under construction. The IPS-MA method is based on eight principles: 1) Sherpa is the patient’s advocate, not an authority or a healthcare provider; 2) the process is led by the individual’s goals and focus is on patient resources; 3) assistance is flexible, without time limits, and responsive to the needs of the patient; 4) the goal is competitive employment or education, without pre-vocational training; 5) the belief is that returning to work is possible despite a mental illness, but therapeutic recommendations in terms of postponement are acknowledged; 6) liaison with healthcare and social workers ensures a coordinated service; 7) a meaningful and realistic career plan will be developed and evaluated continuously after job start; and 8) Sherpa is an interdisciplinary team, which will be reflected in the assistance of each individual.

Five basic services comprise IPS-MA:

1) *Individualised mentor support based on psychiatric knowledge*. Sherpa mentors all have a background as professionals in mental health services. In cooperation with the participant, the Sherpa mentor helps develop a plan of action in which resources and problems in social life as well as working life are clarified. The Sherpa mentor supports the participant in how to structure and manage everyday life, renew contact with friends and/or family, prepare important meetings and live a healthy everyday life with the disorder. The Sherpa mentors very often act as lay representatives for the participants at meetings at the local job centres or municipalities.

2) *Coordination of services provided by Sherpa or external providers*. Through their professional skills, Sherpa mentors help avoid lack of coordination and unnecessary waiting time and make sure that all available services are provided. Sherpa mentors have an assertive approach to mental health carers and social workers and thereby ensure that relevant information is distributed between services.

3) *Career counselling*. Professional career counsellors support participants in creating a realistic match between their competences and the demands of the job market. Participants will be given advice on how to write a curriculum vitae and job applications, on job seeking strategies, and help in practicing job interviews and negotiating employment contracts.

4) *Impartial help to clarify private economy* is offered by a consultancy firm, the Settlement [34], run by volunteers. The firm consists of two employees and a group of volunteers with professional backgrounds in economics, law and social counselling.

5) *Contact with employers* to help participants obtain jobs, and keep them.

Participants are provided with a Sherpa mentor who will be their mentor throughout the entire intervention period. The search for job or education will commence as soon as possible. Mentor support will continue for as long as needed after employment or education is started. During the first 6 months, the participant and mentor most often meet once a week for 1 to 1.5 hours on average. After 6 months the number of contacts varies and can be by telephone or email. The number and duration of contacts depend on the needs of the participant. Each mentor has a maximum caseload of 20 participants, half of which have been in Sherpa for more than 6 months.

#### Sherpa team

The Sherpa team is an interdisciplinary team, consisting of six mentors and two career counsellors. Sherpa mentors all have solid experience as health professionals in mental health services and include one nurse, two social workers and three occupational therapists. Career counsellors have worked as career counsellors, or with recruitment or human resources in the private business sector. Sherpa mentors and career counsellors work closely together and share offices.

#### Training and supervision

Newly appointed Sherpa mentors will have a 1-week introduction to working routines, and will attend a 2-day workshop introducing the IPS-MA method. Mentors with experience in the method will conduct the introduction. Team members are furthermore obliged to participate in annual refresher courses.

Team members will have monthly supervision provided by a trained psychologist.

#### The control group

Participants randomised to the control group will receive ‘treatment-as-usual’ as offered by the job centres in Denmark [35]. Services vary according to match group and the participant’s possibilities for social support. Participants receiving sickness benefits must attend their first meeting in the job centre within 8 weeks of sickness leave. Match group 2 participants attend follow-up interviews every 4 weeks, whereas match group 3 participants attend follow-up interviews every 3 months. Participants on social security will attend job-seeking interviews every 3 months.

Participants under the age of 30 have the right and obligation to participate in pre-vocational training after no more than 13 weeks of unemployment. Pre-vocational training has to last for at least 6 months. Young participants must not be without some sort of pre-vocational training for more than 4 weeks. Participants over the age of 30 have the right and obligation to participate in pre-vocational training after no more than 9 months of unemployment.

After an individual evaluation, job centres can offer certain pre-vocational training services: company internship programmes in public or private companies as well as in sheltered workshops, wage subsidy jobs, skill development and guidance, and mentor support (often offered by a colleague who helps the participant adapt to the new workplace regarding norms and social competences). Participants receiving sickness benefits can be provided with gradual return to employment, assistive tools, a personal assistant or reimbursement of sickness benefits to the employer from the first day of sickness leave [35].

### Participant withdrawal

Participants can choose to withdraw from the trial at any time during the intervention period, without it having any consequences for the treatment they will receive, but they will politely be reminded of the importance of their participation. Participants who choose to withdraw from the trial are asked to specify which aspects of the trial they withdraw from: participation in the experimental intervention, participation in the follow-up interviews, use of data collected at central registers, or complete withdrawal including use of already collected data.

### Fidelity

To ensure that the services provided by Sherpa are in concordance with the IPS-MA method, an independent investigator will monitor fidelity to the IPS-MA method twice during the first year of the intervention, and subsequently once every year. Fidelity will be monitored using the IPS-MA Fidelity Scale (unpublished, available through corresponding author) by interviewing participants, mentors, and career counsellors, observing team-meetings and meetings between mentor and participant, as well as examining the individual plans of action and the data management systems used. The IPS-MA Fidelity Scale was developed based on the IPS Fidelity Scale [36]. Core elements important to the IPS-MA method investigated are: caseload, mentors’ and career counsellors’ roles, interdisciplinary team with group supervision, individualised mentor support, development and evaluation of individual plans of action, coordination of services, providing career and economic counselling, focus on rapid search for ordinary employment or education, no time limitations, and individualised support for the participants and their employers, community-based services, assertive engagement and outreach.

### Assessments

Participants will be interviewed and asked to fill in questionnaires at baseline and at follow-up after 12 and 24 months. At baseline, socio-demographic information on education, income base, marital status, number of children and somatic disease will be collected.

To confirm the diagnosis, the Mini International Neuropsychiatric Interview (MINI) [37] is used at baseline. Baseline interviews will always be face-to-face, most often in the participants’ home. Participants will fill in questionnaires at home.

### Outcomes

The primary outcome is competitive employment (including being on rehabilitation benefits, flexible jobs, and wage-subsidised jobs) or education at 24 months. Information about employment and education will be extracted from the DREAM database [38]. The database is administered by The National Labour Market Authority and contains information on employment, sickness leave, and education eligible to state education grant, pre-vocational training, disability pension, social security, and sickness benefits.

Secondary outcomes are: 1) number of days of competitive employment or education; 2) level of symptoms assessed by the Hamilton Depression Scale (HAM-D6) [39,40]; 3) level of symptoms assessed by the Hamilton Anxiety Scale (HAM-A6) [39,41]; 4) level of functioning assessed by The Global Assessment of Functioning (GAF) [39,42,43]; and 5) level of health-related quality of life by The WHO-Five Well-being Index(WHO-5) [39]. Secondary outcomes are assessed after 12 and 24 months.

Exploratory outcomes are: competitive employment (including being on rehabilitation benefits, flexible jobs and wage subsidy jobs) or education at 12 months, re-assignment from Match group 2 or 3 to Match group 1, attending company internship programs in public or private companies as well as in sheltered workshops, and information extracted from the DREAM database. Manic symptoms are assessed by the Bech-Rafaelsen Mania Scale (MAS) [39,44]. Social performance regarding four domains (socially useful activities, personal and social relationships, self-care and disturbing and aggressive behaviour) is assessed by The Personal and Social Performance (PSP) [45,46]. The Sheehan Disability Scale [47] measures functional level regarding social relationships, work, spare time and family. Health-related quality of life in terms of psychological well-being is assessed by the WHO-5 [39,48] and empowerment by the Empowerment Scale [49]. The Changes Questionnaire [50] will be used to assess how motivated participants are as to seeking employment or education. The Client Satisfaction Questionnaire [51] assesses satisfaction with treatment and the EQ-5D (EuroQol) [52] assesses health-related quality of life. The latter of the two will be used in a future health-related cost-benefit analysis. All scales and questionnaires used for measuring outcomes are validated scales [37,39-47,49-52].

#### Register-based information

Information on vital status, use of mental health services, both as in- and outpatient, number of days of admission, sickness absence and use of social benefits will be gathered from the DREAM database or the Danish Psychiatric Case Register (DPCR) [53]. DPCR is the patient-registry system used by the mental health services in Denmark; it contains information on all hospital admissions, number and duration, outpatient contacts and deaths.

An overview of all data collected and the source of collection is shown in Table 1.

**Table 1:** Data collection at baseline and follow-up

**Source of collection**	**Assessment**	**Baseline**	**12 months follow-up**	**24 months follow-up**
Interview	Hamilton Depression Scale (HAM-D6)	x	x	x
	Hamilton Anxiety Scale (HAM-A6)	x	x	x
	Bech-Rafaelsen Mania Scale (MAS)	x	x	x
	Personal and Social Perfomance scale (PSP)	x	x	x
	Global Assessment of Functioning (GAF)	x	x	x
	Suicidal ideation	x	x	x
Self report	Sheehan Disability Scale (SDS)	x	x	x
	Quality of life (WHO-5)	x	x	x
	Empowerment Scale	x	x	x
	Changes Questionnaire	x	x	x
	Client Satisfaction Questionnaire (CSQ)	x	x	x
	Health-related quality of life EQ-5D (EuroQol)	x	x	x
Hospital records	Number of hospital admissions		x	x
	Length of hospital admissions		x	x
	Use of outpatient services		x	x
	Death (all causes)		x	x
	Suicide		x	x
Dream/interview	Sociodemographic information	x	x	x
Dream	Labour market affiliation	x	x	x
Dream/interview	Civil status	x	x	x
DPCR	First contact with mental health care	x		
Dream/interview	Children	x	x	x
Dream/interview	Education	x	x	x
Dream/interview	Cohabitation status	x	x	x
DPCR	Use of mental health service		x	x
Dream	Number of sick days		x	x
Dream	Use of social benefits		x	x
Self report	Treatment and use of other service from the social and healthcare sector		x	
Self report	Service provided by Sherpa		x	

DPCR, The Danish Psychiatric Case Register.

All data will be handled in accordance with the Danish Data Protection Agency.

### Training and inter-rater reliability

Three assessors conduct the interviews: Britt Reuter Morthorst (BM), Marie Lønberg Hansen (MLH) and LH. BM and LH have a masters in health science, and MLH in public health science. BM has 15 years experience as a nurse in mental health, and is an experienced assessor. Assessors have all received the necessary training in the relevant instruments. All assessors have participated in joint ratings for HAM-D and HAM-A with PB. Regarding the MINI, MAS, PSP and GAF, at least seven joint ratings have been conducted in order to ensure inter-rater reliability.

For the evaluation of inter-rater reliability the intra-class coefficient was used [54]. The level of significance was a coefficient of 0.70 or higher.

LH has participated in joint HAM-D6 and HAM-A6 rating sessions with PB. In total, 28 joint sessions between PB and LH were evaluated and for HAM-D6 the intra-class coefficient was 0.81 (*P* < 0.001). Together LH and BM have seen seven patients in joint training sessions; intra-class correlations were: PSP = 0.92, GAF-Functioning = 0.84, GAF-Symptoms = 0.75.

### Power and sample size

We have been unable to find data on how many people actually return to employment or education with traditional pre-vocational training after anxiety or an affective disorder in either the Danish or the national literature. Therefore, we have leaned towards the findings in OPUS, a programme in which young people with schizophrenia receive early intensive treatment for 24 months. In OPUS it was found that 40% returned to employment or education versus 32% in the control group (Merete Nordentoft, personal communication). Based on this knowledge, we conservatively estimate that 30% will regain employment or education following traditional pre-vocational training.

Across a broad range of studies of severe mental illness and IPS versus traditional pre-vocational training, studies show that approximately 50% more of the participants in the IPS groups regain employment compared to the control groups [15]. We therefore expect to find that 50% more of the participants in the Sherpa group compared to the control group will regain employment or education, and have estimated the true difference in the experimental and control group to be 15%-points; hence, 45% of the participants in the Sherpa group will regain employment. To be able to reject the null hypothesis that the proportion of participants who regain employment or education in the experimental and control group is equal with a probability (power) of 80%, 162 participants will be required in each group (total 324). The Type I error probability associated with the test of this null hypothesis is 5%. We also estimated the sample size using a power of 90%. This resulted in a total of 434 participants (2 × 217). We therefore plan to recruit a minimum of 324 participants and, in order to reduce the risk of type II error, we will aim to recruit up to 434 participants, if possible, in the 2-year recruitment period. Power and sample size calculations have been made using the PS Power and Sample Size Calculations program version 3.0.14 [55,56].

The power for the secondary outcomes has been estimated based on a number of 162 participants in each group (Table 2). Since it has not been possible to find studies or trials similar to our trial regarding patient group or method, expected effect size concerning number of days in employment or education has been conservatively estimated. The studies found [1,9,19,21,22,57-59] did not find any difference between groups after 12 months considering GAF-F, WHO-5, HAM-D6 or HAM-A6. If we find a difference between groups, we want it to be clinically relevant; therefore, the effect sizes equals the clinically relevant difference.

**Table 2:** Power calculations for secondary outcomes, calculated from a sample size of 324 participants

**Measure**	**Mean difference**	**Standard deviation of the pooled mean**	**Type I error**	**Reference**	**Power**
No of days of competitive employment of education at 12 months	60 days	150 days	5%	Kin W 2008 [21], Burns 2007 [14]	95%
GAF-F	5	15	5%	Hoffmann 2011 [19], Howard 2010 [58]	85%
WHO-5	10	19	5%	Latimer 2006 [22], Burns 2009 [14]	99%
HAM-D6	2	4	5%	Wang 2007 [10], Lexis 2011 [9], Brouwers 2006 [1], Van Oostrom 2010 [59]	99%
HAM-A6	2	4	5%	Wang 2007 [10], Lexis 2011 [9], Brouwers 2006 [1], Van Oostrom 2010 [59]	99%

GAF, Global Assessment of Functioning; HAM-A6, Hamilton Anxiety Scale; HAM-D6, Hamilton Depression Scale; WHO-5, WHO-Five Well-being Index.

### Statistical analyses

Data analyses will be based on the intention-to-treat principle, which means that data will be included in the group to which the participant was randomised, regardless of intervention received. Data will be analysed using the IBM SPSS Statistics version 20 for Windows.

To assess homogeneity of the two groups at baseline, demographic data such as age, gender, marital status, education level, support (social benefits, social security and so forth), diagnosis and Match group at baseline will be presented.

Dichotomous outcomes will be analysed using logistic regression. For primary and secondary outcomes, an unadjusted analysis of the effect of the Sherpa method as an add-on to treatment-as-usual versus exclusively treatment-as-usual will be carried out, as well as an analysis adjusted for stratification variables (diagnosis and Match group). Multiple multivariate imputations will be used to impute a distribution of missing values.

Continuous outcomes will be analysed in a mixed model with repeated measurements. This model is based on the assumption that data are missing at random or missing completely at random [60].

### Feasibility

In 2010, 11,712 inpatient visits were registered in the Mental Health Care Centres of the Capital Region of Denmark, not including emergency wards [61]. During the same period of time there were 4,538 first-time psychiatric emergency ward visits in the Capital Region of Denmark [61]. Based on these figures we find it realistic to include a minimum of 324 participants from 1 October 2011 until 31 December 2013.

Each mentor has a maximum caseload of 20 participants per year; six mentors are currently engaged in Sherpa. Thus it is also realistic regarding the capacity of Sherpa mentors to include and complete the intervention for 162 participants in 3 years.

### Ethical considerations

All participants in this trial, randomised to experimental as well as control group, are offered treatment according to best practice. The trial will follow international ethical guidelines of informed consent in clinical trials. Participants will receive written and verbal information about the trial so as to be able to give an informed consent. Consent has to be given verbally and in writing. Participation is voluntary, and participants can withdraw their consent at any time during the trial without it having any consequences for their treatment. Previous trials have not found any risks or adverse reactions to the supported employment intervention [19,31,62,63]. If any of the participants present suicidal ideations, the mentor and assessor will make sure that they can be distracted from these thoughts, have a crisis plan, are not alone after the interview and, if in doubt of any of the above, they will offer to follow the participant to the psychiatric emergency ward.

The trial protocol was submitted to the Regional Ethics Committees of the Capital Region for review (journal no: H-2-2011-FSP20). The committee assessed the protocol to be exempt from formal approval, since it is not a biomedical trial. The trial has been reported to the Danish Data Protection Agency (RHP journal no: 2007-58-0015, local journal no: RHP-2011-20) and has been registered at http://www.clinicaltrials.gov identifier: NCT01721824.

## Trial status

The trial is on-going; 290 participants have been randomised, and recruitment continues until 31 January 2014.

## Discussion

The IPS-MA method is based on a 1-year pilot study and the evidence supporting IPS in other countries. To our knowledge this is the first trial investigating the effect of a supported employment intervention when provided to people with a recently diagnosed affective disorder or anxiety disorder, an area with only sparse knowledge about effective interventions. A strength of the study is the centralised computer-based randomisation which ensures an adequate generation of the allocation sequence and adequate allocation concealment. The use of blinded outcome assessors for the primary outcome and the fact that it is a register-based outcome as well as the use of intention-to-treat analysis decreases the risk of biased effect estimates. The trial is registered at http://www.clinicaltrials.gov, which helps preventing selective and incomplete outcome reporting. The primary outcome is register-based, which ensures almost complete follow-up due to the comprehensiveness of Danish registers.

The fact that we monitor fidelity to the IPS-MA method on a yearly basis is another strength of this trial. We do so to ensure that mentors and career counsellors are true to the method.

A limitation to this trial is that we are not able to blind participants, mentors or carers. Some might argue that it is difficult to sustain the blinding of the assessor during follow-up, and this is certainly a risk of bias. Should blinding be violated, a second assessor will complete the follow-up interview.

Even though participants are recruited from mental health centres throughout the Capital Region of Denmark, and should be fairly representative of the population in the region, we may have a reduced external validity. As it is the staff at the mental health centres that identify eligible participants, not everybody with an affective disorder or anxiety disorder eligible might have been asked to participate; patients are not systematically screened for eligibility.

Due to differences in labour markets and well-fare systems, results may not be directly generalisable to other countries.

### Impact of the results

The results of this trial will add to the limited knowledge regarding vocational rehabilitation for people with recently diagnosed anxiety or affective disorders. If potential positive results can be confirmed in other trials, the IPS-MA method can be implemented at the job centres nationwide, and would probably prevent a large number of disability pensions and long-term sickness absences with major benefits to society and patients.

### IPS-MACompeting interests

LH’s PhD is exclusively founded by the Obel Family Foundation. Due to administrative convenience, PhD student LH was formally employed by Sherpa from 1 June 2011 until 31 August 2013. LH has throughout the entire period been working at the Research Unit at Mental Health Centre Copenhagen, where she is now employed. Managerial responsibility and supervision lie with LFE and PB. Sherpa has had no role in the trial design, and will have no role in collection of data, analysis of data, data interpretation, or in publication of data from the trial. None of the other authors have any competing interest.

## Abbreviations

DPCR: Danish psychiatric case register; GAF: Global assessment of functioning; HAM-A6: Hamilton anxiety scale; HAM-D6: Hamilton depression scale; IPS: Individual placement and support; IPS-MA: IPS-modified, early intervention for people with mood and anxiety disorder; MAS: The Bech-Rafaelsen mania scale; MINI: Mini international neuropsychiatric interview; PSP: Personal and social performance; WHO-5: WHO-Five well-being index.

## Competing interests

The authors declared that they have no competing interests.

## Authors’ contributions

LFE conceived the trial, participated in the planning and design, and read and critically revised the manuscript for important intellectual content. LH participated in the planning and design of the trial, conducted the research interviews, drafted the manuscript and, along with LFE, critically revised it for important intellectual content. MN participated in the planning and design of the trial, and has read and critically revised the manuscript. JL participated in the planning and design of the trial, and has read and critically revised the manuscript. PB participated in the planning and design of the trial, was responsible of the training of the assessors, and has read and critically revised the manuscript. All authors read and approved the final version of the manuscript.
